# Laboratory versus daily life gait characteristics in patients with multiple sclerosis, Parkinson’s disease, and matched controls

**DOI:** 10.1186/s12984-020-00781-4

**Published:** 2020-12-01

**Authors:** Vrutangkumar V. Shah, James McNames, Martina Mancini, Patricia Carlson-Kuhta, Rebecca I. Spain, John G. Nutt, Mahmoud El-Gohary, Carolin Curtze, Fay B. Horak

**Affiliations:** 1 Department of Neurology, Oregon Health & Science University, 3181 SW Sam Jackson Park Road, Portland, OR 97239-3098 USA; 2 Department of Electrical and Computer Engineering, Portland State University, Portland, OR USA; 3 APDM Wearable Technologies, Portland, OR USA; 4 Veterans Affairs Portland Health Care System, Portland, OR USA; 5 Department of Biomechanics, University of Nebraska At Omaha, Omaha, NE USA

**Keywords:** Gait, Free-living, Parkinson’s disease, Multiple sclerosis, Laboratory

## Abstract

**Background and purpose:**

Recent findings suggest that a gait assessment at a discrete moment in a clinic or laboratory setting may not reflect functional, everyday mobility. As a step towards better understanding gait during daily life in neurological populations, we compared gait measures that best discriminated people with multiple sclerosis (MS) and people with Parkinson’s Disease (PD) from their respective, age-matched, healthy control subjects (MS-Ctl, PD-Ctl) in laboratory tests versus a week of daily life monitoring.

**Methods:**

We recruited 15 people with MS (age mean ± SD: 49 ± 10 years), 16 MS-Ctl (45 ± 11 years), 16 people with idiopathic PD (71 ± 5 years), and 15 PD-Ctl (69 ± 7 years). Subjects wore 3 inertial sensors (one each foot and lower back) in the laboratory followed by 7 days during daily life. Mann–Whitney U test and area under the curve (AUC) compared differences between PD and PD-Ctl, and between MS and MS-Ctl in the laboratory and in daily life.

**Results:**

Participants wore sensors for 60–68 h in daily life. Measures that best discriminated gait characteristics in people with MS and PD from their respective control groups were different between the laboratory gait test and a week of daily life. Specifically, the toe-off angle best discriminated MS versus MS-Ctl in the laboratory (AUC [95% CI] = 0.80 [0.63–0.96]) whereas gait speed in daily life (AUC = 0.84 [0.69–1.00]). In contrast, the lumbar coronal range of motion best discriminated PD versus PD-Ctl in the laboratory (AUC = 0.78 [0.59–0.96]) whereas foot-strike angle in daily life (AUC = 0.84 [0.70–0.98]). AUCs were larger in daily life compared to the laboratory.

**Conclusions:**

Larger AUC for daily life gait measures compared to the laboratory gait measures suggest that daily life monitoring may be more sensitive to impairments from neurological disease, but each neurological disease may require different gait outcome measures.

## Background

Gait impairments are very common in patients with neurological disorders, leading to an elevated risk of falls and reduced quality of life [1–3]. Quantitative gait assessment can often determine the problem(s) underlying the gait impairment and then can be useful to test an efficacy of a new intervention. However, until recently, quantitative gait assessments were limited to specialized laboratories, under well-controlled conditions. Although laboratory gait assessments provide information about gait under controlled conditions, they may not reflect actual, functional gait performance during daily activities [4–6]. It is likely that increased attention to the walking task and awareness of being observed (Hawthorne effect) minimizes gait impairments in the laboratory while divided attention, cluttered environments, varied sensory conditions, and fatigue may result in worse gait impairments during daily life. Thus, gait assessment in the laboratory reflects a person’s capacity (what a person can do), whereas gait during daily life reflects a person’s functional performance (what a person is actually doing) [5, 6]. This understanding is important while conducting research as we might see only optimal performance during clinical or laboratory visits and daily performance may be worse than what is observed in these prescribed tasks. As a result, clinicians might underestimate potential gait impairments related to daily life functional abilities.

Further, specific types of mobility impairments differ depending upon the neurological disorder. For example, gait in people with MS is characterized by reduced endurance, spasticity, and ataxia, whereas gait in people with PD is characterized by bradykinesia, shuffling, rigidity, freezing, and difficulties turning [7–10]. Slowed gait speed is very common with any neurological disorder or age [11]. However, slow gait is a general, universal characteristic of impaired mobility and hence, may not be the most specific nor discriminative mobility impairment in each neurological disorder.

Recently, the use of wearable technology has made it feasible to quantify gait in the laboratory and during daily life [12–30]. Several studies have compared the quality of mobility in the laboratory with daily life walking bouts [5, 14, 31–33]; however, these studies did not compare similar gait bout lengths in the two environments (laboratory versus daily life) except for the one recent study in children with cerebral palsy [32]. Specifically, Del Din et al. [14] compared 10-m walking bout in the laboratory to all walking bouts during daily life in people with PD, Storm et al. [31] compared 15-m and 1-min walking bout in the laboratory to all bouts with < 50 steps, between 51 to 100 steps and > 100 steps in people with MS. Hillel et al. [5] compared 1-min laboratory walking bout to daily life walking bout of 30-s only in people with PD. Shema-Shiratzky et al. [33] compared the first 30-s of 1-min laboratory walking bout to daily life walking bout of 30-s and more in people with MS. Matching gait bout length is important because many gait measures change with the duration of a walking bout [14], [34]. In addition, people very seldom, if ever, walk for more than 1-min continuously or in a straight line for over 10-m during daily life like they do in laboratory tests [4, 5, 14, 31]. Thus, comparisons of strides taken from long, steady-state gait in the laboratory with all the strides measured in daily life are confounded by differences in gait bout length. Hence, in this study, we focused on short walk test in the laboratory and compared the gait characteristics in the laboratory to similar short walking bouts during daily life.

In this study, we aimed to identify a set of gait measures that best discriminated gait characteristics from a single short walk gait test in the laboratory between people with MS and their age-matched healthy control subjects (MS-Ctl), and between people with PD and their older, age-matched healthy control subjects (PD-Ctl) and compared those gait quality measures to a week of daily life gait quality measures from a similar short bout length using wearable sensors. We investigated whether gait measures that best discriminate gait impairments in MS and PD versus their respective control cohorts during laboratory assessments remain the same during a week of daily life assessment. Further, we investigated the group differences between laboratory and daily life gait measures for MS, MS-Ctl, PD, and PD-Ctl. We hypothesized that: (1) different gait measures would best discriminate PD vs. PD-Ctl and MS vs. MS-Ctl in the laboratory and daily life, and (2) daily life gait would be more discriminative than laboratory measures for both neurological groups. Recent studies have shown that the laboratory gait measures do not reliably reflect daily life gait measures in people with PD and MS [5, 14, 33]. Hence, we expected different gait measures would discriminate in the laboratory and daily life for both neurological groups. We also expected that daily life would provide a more complete picture of functional performance in a complex environment, such that group differences would be more evident in daily life compared to laboratory gait measures. Further, we explored which specific gait measures were the most discriminative for the PD and the MS groups, both in the laboratory and daily life.

## Methods

### Participants

We recruited people with MS, age-matched MS-Ctl, people with PD, and age-matched PD-Ctl for this study which is the part of a larger, longer study to identify gait measures that predict prospective falls in MS and PD. Our cohort with MS was younger than the cohort with PD, so we recruited younger and older control groups for comparison. Inclusion and exclusion criteria were the same as described in Shah et al. [35]. Specifically, inclusion criteria for PD were a diagnosis of idiopathic Parkinson’s disease from movement disorders neurologist with the United Kingdom Parkinson’s disease Society Brain Bank criteria, Hoehn & Yahr scores of II-IV, and complaints about gait. Inclusion criteria for MS were a diagnosis of relapsing–remitting MS confirmed by a neurologist specialist, a mild-to-moderate MS-associated disability (patient-reported EDSS score ≤ 6.0), and complaints about gait. Exclusion criteria for all subjects included the inability to follow protocol instructions, other factors affecting gait such as musculoskeletal disorders, uncorrected vision or vestibular problems, or inability to stand or walk in the home without an assistive device. The experimental protocol was approved by the Institutional Review Board of the Oregon Health & Science University (eIRB #15578). The experimental protocol was carried out in accordance with the institution’s ethical committee, and all the participants provided informed written consent.

### Laboratory data collection

**Fig. 1:**
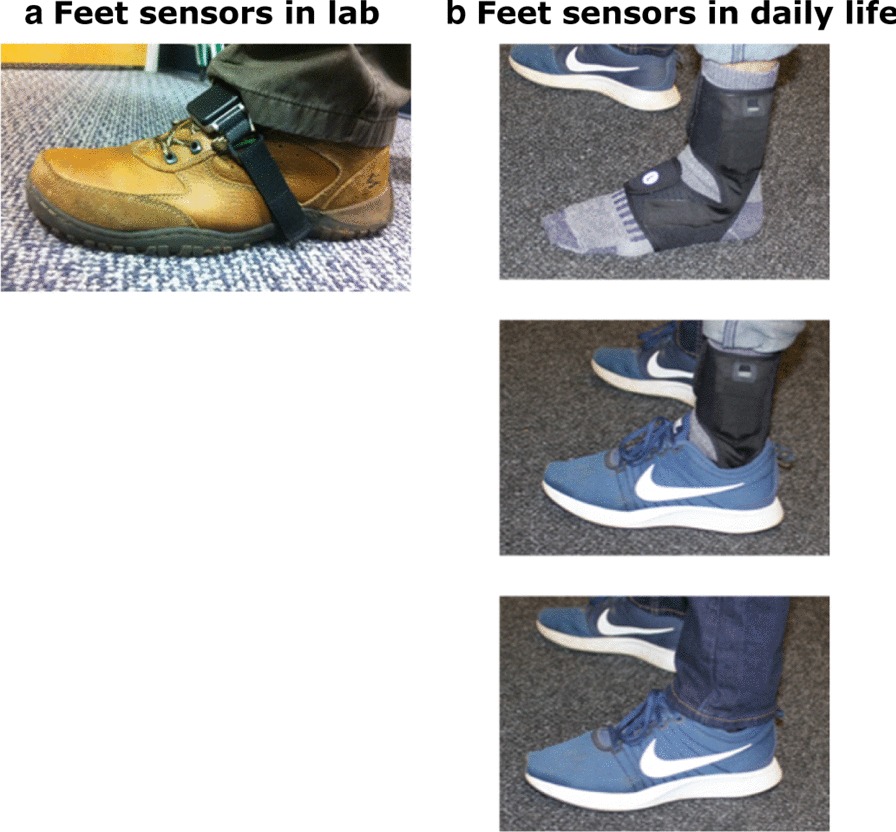
Sensor placement on feet. **a** Opal inertial sensor on foot for laboratory testing. **b** Instrumented sock prototype for daily life data collection. Note inertial sensor located over lateral metatarsals and battery located just above lateral malleolus

In the laboratory, subjects were asked to wear 3 inertial sensors (Opals by APDM Wearable Technologies, Portland, OR, USA; Fig. 1a); one sensor on top of each foot, and one over the lower lumbar on an elastic belt. Each Opal sensor includes a tri-axial accelerometer, gyroscope, and magnetometer with a sampling rate of 128 Hz. The Opal is lightweight (22 g), has a battery life of 16 h, and includes 8 GB of storage, that can record over 30 days of data. All the subjects performed the Instrumented Stand and Walk test (ISAW) [36]. The ISAW consists of standing quietly for 30 s, followed by a verbal instruction to initiate gait, walk 7 m, turn 180 degrees after crossing a line on the ground, and return to the initial starting position [36]. The ISAW test was designed to measure postural sway, step initiation, gait, and turning, all in one short test. Individuals with MS completed estimates of the severity of MS with Patient-Reported Expanded Disability Status Scale (PREDSS) [37, 38], walking ability with Multiple Sclerosis Walking Scale (MSWS-12) [39], and fatigue with Modified Fatigue Index Scale (MFIS) [40]). PD severity was assessed by a certified researcher using the Movement Disorders Society-Unified Parkinson’s Disease Rating Scale (MDS-UPDRS), Part III [41]). All subjects were tested in the laboratory 1 h after taking their regular medication intake (ON-medication state), because most of the time, subjects attempt to be in the ON-medication state in daily life.

### Daily life gait data collection

Subjects were asked to wear instrumented socks (prototype developed by APDM Wearable Technologies, Portland, OR, USA; Fig. 1b) on each foot, and one Opal sensor over the lower lumbar area with an elastic belt. They wore the sensors for at least 8 h/day for a week of daily life. To reduce the burden on participants of trying to securely attach the Opal monitors to the outside of their shoes, APDM Wearable Technologies designed an instrumented sock that wraps around the participant’s foot and ankle with the inertial sensor inserted into a small, lightweight pocket in the foot area. The inertial sensor within the sock is located on the dorsum of the foot like the Opal sensors worn in the laboratory, The main unit containing the battery is located in a second pocket just above the lateral malleolus. To maximize fit, the socks come in different sizes, and the Velcro attachment around the foot and ankle is adjustable to ensure that a snug fit and that the sensor does not move on the foot while being worn. Thus the inertial system fits into the instrumented sock for ease of application and safe, unobtrusive use. The instrumented socks and Opals have the same inertial sensors with the same sensor specifications but different form factor. The instrumented socks are synchronized with the Opal worn on the lumbar area. This allowed subjects to comfortably wear the instrumented socks in their shoes or slippers, and without a visually-distracting external strap attachment. It also eliminated the task of securing the monitors on the shoes, especially important for people (such as people with PD), who have difficulty with fine motor movements, such as tying shoelaces. The subjects removed the socks and the belt at night to recharge the batteries. Data were stored in the 8 GB internal memory of the Opals and socks. Subjects returned instrumented socks either by mail using a pre-paid box after completion of a week of data collection or to a research assistant who met subjects at their homes. Data were uploaded to a secure cloud-based database upon return of the devices and downloaded to a local computer for further processing using the same gait algorithms for laboratory and daily life gait (after identification of appropriate length gait bouts).

### Measures of gait

The algorithms used for extracting spatial and temporal measures of gait and turning are identical for laboratory and daily life gait and have been detailed previously [35, 42]. In addition, the daily life algorithm first searches for possible bouts of walking using a time-domain approach to inertial sensor data from the feet and for turns based on yaw rotational orientation of the pelvis. Second, individual steps are combined into potential bouts of walking, as long as the duration from one step to the next step is no longer than 2.5 s. Finally, each possible bout that contains at least 3 steps and is at least 3 s in duration is processed with the commercial gait analysis algorithms included in Mobility Laboratory (APDM Wearable Technologies, Portland, Oregon) [36, 43, 44]. Our gait analysis algorithm uses the Unscented Kalman Filter to fuse information from the accelerometers, gyroscopes, and magnetometers to precisely estimate the orientation and position trajectory of each foot between quiet stance periods [45, 46]. This approach reduces the problem of tracking over a long period of time.

To compare between in-laboratory and daily life gait measures, we used only gait bouts that had a similar number of strides during the ISAW test in the laboratory and during free-living daily activities (4–15 strides in about). Specifically, we calculated number valid of strides for the ISAW test in the laboratory for all participants and found that it ranged between 4 to 15 strides, so for the bout analysis in daily life we used the same range (4–15 strides) corresponding to the stride range observed in the laboratory ISAW test. In total, we chose 13 gait measures (see Fig. 3 and Additional file 1: Table S1) that are commonly used to characterize gait in the laboratory and grouped them into three aspects of gait for simplicity: 3 upper-body, 5 spatial, and 5 temporal measures of gait [47]. We avoided comparing the coefficient of variation measures as it is affected by the environment and by bout length [48], and also the number of strides in a bout for our analysis was less than the minimum number of strides required to accurately calculate a coefficient of variation (20 strides) [49, 50].

### Statistical analysis

The normality of data was determined with Shapiro–Wilk tests and parametric analysis was used, unless otherwise stated. Independent t-tests or Mann–Whitney U tests (if not normally distributed) were used to compare between-group differences in subject characteristics, adherence, and total weekly walking bouts. As not all gait quality measures in the laboratory and during daily life were normally distributed, to be consistent, non-parametric test (Mann–Whitney U test) was used to compare differences between groups for all gait measures (i.e., MS vs MS-Ctl and PD vs PD-Ctl). In addition, we computed Receiving Operating Characteristics (ROC) and calculated the Area Under Curve (AUC) [51] to discriminate gait measures in people with MS from MS-Ctl and people with PD from PD-Ctl, and ordered measures from the highest to lowest AUC value. Paired Wilcoxon tests were used to compare the laboratory and daily life gait measures for MS, MS-Ctl, PD, and PD-Ctl. All statistical analysis was performed using R Version 1.1.456 software. The statistical significance was set to *p* < 0.05, and we used threshold of p ≤ 0.004 based on the Bonferroni’s correction (due to 13 multiple comparisons) just to prioritize gait measures.

## Results

### Group characteristics and adherence

**Fig. 2:**
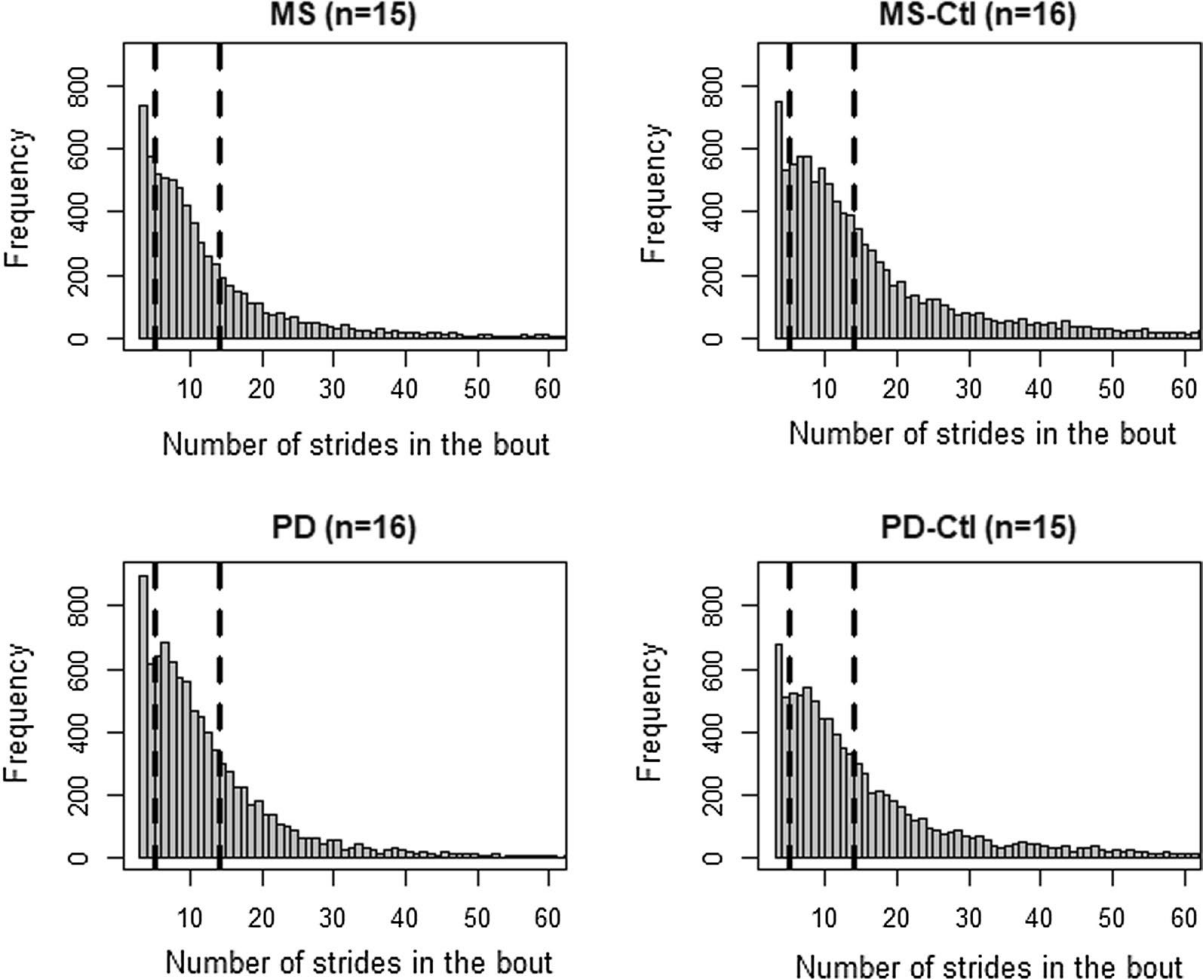
Histogram of the grouped total of bout during daily life for each group. The dashed line indicates the range of the number of strides in the bout used for the analysis

Fifteen people with MS, 16 age-match MS-Ctl, 16 people with PD (Hoehn and Yahr stage with II (n = 26), III (n = 2), and IV (n = 1)), and 15 age-matched PD-Ctl participated in the study. Table 1 shows the demographics and activity characteristics of subjects who participated in this study. Age, height, and weight were similar between the MS and MS-Ctl and between the PD and PD-Ctl groups. Adherence to the weekly recordings for each subject group was similar with 60.19 ± 11.02 (mean ± SD) hours in MS, 64.15 ± 9.59 h in MS-Ctl, 67.66 ± 12.53 h in PD, and 64.67 ± 10.13 h in PD-Ctl of daily life data. The histogram in Fig. 2 illustrates the number of strides in each bout during daily life, and it is evident that the stride range (4–15 strides) in a bout considered for the analysis in this study captures the major portion of participants’ daily activity.

**Table 1:** Demographics, adherence, and weekly activity of each group

	MS (N = 15)		MS-Ctl (N = 16)		*p*	PD (N = 16)		PD-Ctl (N = 15)		*p*
	Mean	SD	Mean	SD		Mean	SD	Mean	SD	
Age (yrs)	48.73	10.13	44.63	10.73	0.29	70.5	5.11	68.6	6.50	0.44
Height (m)	1.70	0.09	1.67	0.08	0.98	1.73	0.09	1.73	0.12	0.84
Weight (kg)	72.36	21.03	65.43	11.55	0.58	75.38	14.02	76.99	17.90	0.70
PREDSS (#)	4.27	0.70								
MSWS (#)	29.00	8.91								
MFIS (#)	38.80	17.01								
UPDRS III ON (#)						30.44	10.76			
H & Y Stage (#)						2.25	0.56			
Total hours (#)	60.19	11.41	64.15	9.91	0.32	67.66	12.95	64.67	10.49	0.55
Total Bouts (#)	467.67	183.41	656.69	225.71	0.02	585.50	300.03	619.73	215.04	0.71
Bouts used for analysis (#)	228.33	113.40	282.88	118.96	0.20	292.25	145.99	272.33	95.73	0.91

*MS* multiple sclerosis, *MS-Ctl* age-matched control subjects corresponding to MS, *PD* Parkinson’s disease, *PD-Ctl* age-matched control subjects corresponding to PD, *PREDSS* Patient-Reported Expanded Disability Status Scale, *MSWS* Multiple Sclerosis Walking Scale, *MFIS* Modified Fatigue Index Scale, *UPDRS* Unified Parkinson’s Disease Rating Scale, *H & Y* Hoehn and Yahr

### Laboratory versus daily life gait measures discriminating gait in MS from MS-Ctl

**Fig. 3:**
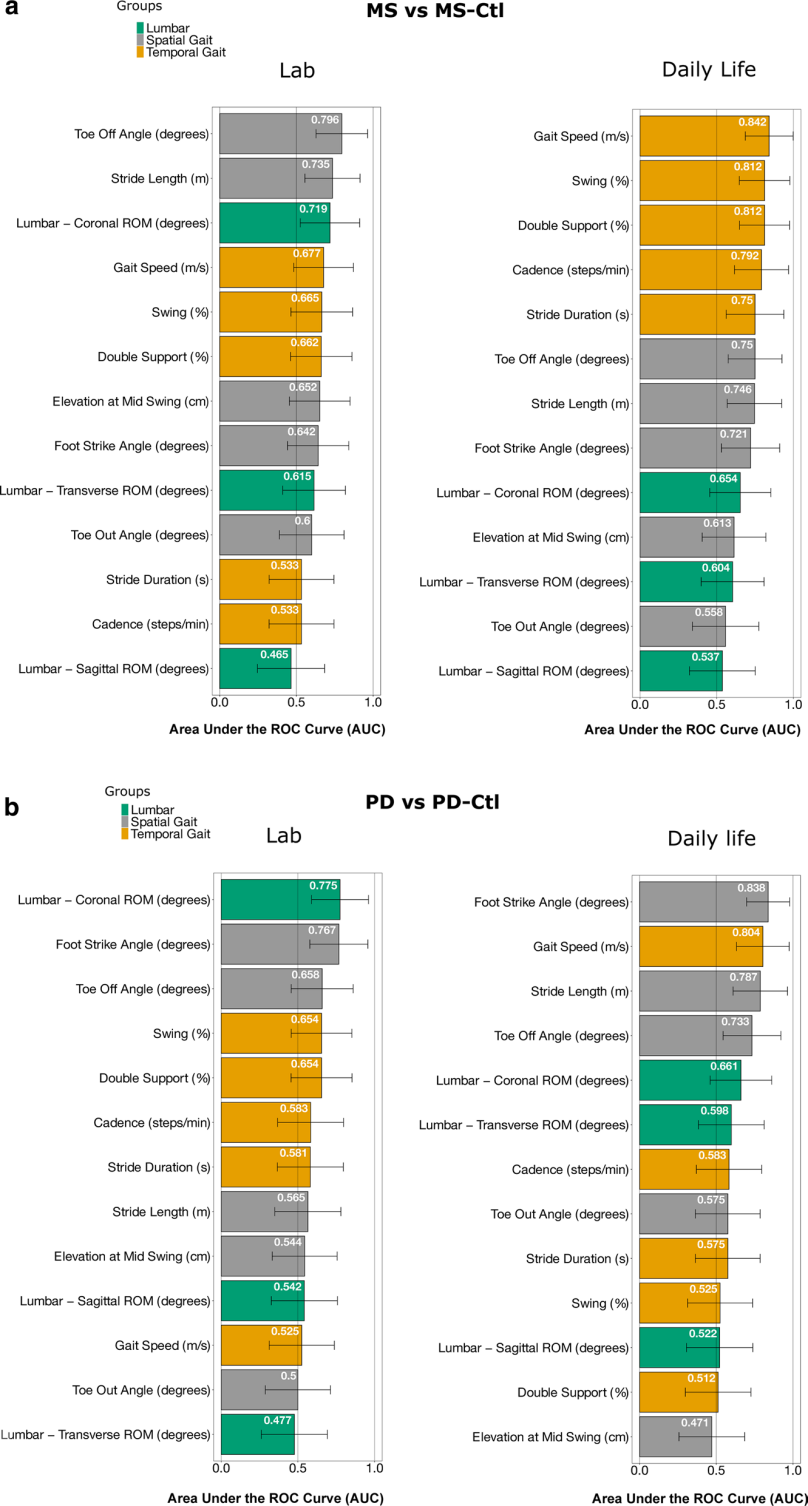
AUC (in descending order with 95% CI) for each mobility measure discriminating **a** people with MS from MS-Ctl, and **b** people with PD from PD-Ctl. The color-coding scheme is based on three aspects of gait

Measures discriminating gait characteristics in people with MS from MS-Ctl in laboratory versus daily life were different (see Table 2 and Fig. 3a). Specifically, the toe-off angle was the most discriminative in the laboratory (p = 0.004; AUC [95% CI] = 0.796 [0.628–0.964]) whereas gait speed was in daily life (p = 0.001; AUC = 0.842 [0.686–0.998]). Stride length was the second best discriminative measure in the laboratory (p = 0.027; AUC = 0.735 [0.556–0.915]) whereas the duration of swing phase as percent of gait cycle was in daily life (p = 0.002; AUC = 0.812 [0.648–0.977). Furthermore, more gait measures discriminated MS from MS-Ctl in daily life (n = 3) compared to the laboratory (n = 1), after the Bonferroni’s correction (13 multiple tests).

**Table 2:** Comparison of gait measures between MS and control groups in the laboratory and during daily life

Gait measures	Laboratory					Daily Life				
	MS-Ctl (N = 16)		MS (N = 15)		Wilcox *p-*value	MS-Ctl (N = 16)		MS (N = 15)		Wilcox *p-*value
	Mean	SD	Mean	SD		Mean	SD	Mean	SD	
Cadence (steps/min)	114.11	9.69	112.59	10.9	0.770	*106.92*	*8.36*	*99.57*	*7.68*	*0.005*
Double Support (%)	20.3	2.65	22.06	4	0.129	*22.67*	*3.55*	*26.85*	*3.61*	*0.002*^*†*^
Elevation at Mid Swing (cm)	0.81	0.32	1.07	0.47	0.155	2.98	0.69	2.71	0.53	0.299
Gait Speed (m/s)	1.11	0.15	1	0.16	0.097	*1*	*0.17*	*0.8*	*0.13*	*0.001*^*†*^
Foot Strike Angle (degrees)	17.97	3.34	15.65	4.68	0.188	*19.38*	*2.64*	*16.25*	*4.16*	*0.037*
Toe Off Angle (degrees)	*36.31*	*3.44*	*31.85*	*3.97*	*0.004*^*†*^	*27.97*	*3.83*	*23.96*	*4.01*	*0.017*
Stride Duration (s)	1.06	0.09	1.08	0.11	0.767	*1.17*	*0.1*	*1.25*	*0.1*	*0.017*
Stride Length (m)	*1.16*	*0.11*	*1.07*	*0.13*	*0.027*	*1.1*	*0.16*	*0.95*	*0.15*	*0.019*
Swing (%)	39.84	1.34	38.91	2.1	0.123	*38.81*	*1.85*	*36.58*	*1.85*	*0.002*^*†*^
Toe Out Angle (degrees)	9.57	4.45	11.13	6.16	0.358	17.93	8.47	18.89	11.51	0.599
Lumbar—Coronal ROM (degrees)	*8.47*	*1.45*	*7.22*	*1.91*	*0.040*	7.45	1.72	6.31	1.72	0.151
Lumbar—Sagittal ROM (degrees)	6.18	2.26	6.15	1.86	0.752	6.87	1.15	6.87	1.15	0.740
Lumbar—Transverse ROM (deg)	8.75	2.85	9.79	2.57	0.286	16.62	2.87	16.62	1.72	0.338

Italics indicates p < 0.05, and ^†^ indicates if the p-value ≤ 0.004 level of significance after Bonferroni correction (that is, 0.05/13 = 0.004)

### Laboratory versus daily life gait measures discriminating gait in PD from PD-Ctl

Most of the measures discriminating gait characteristics in people with PD from PD-Ctl in laboratory versus daily life were different (see Table 3 and Fig. 3b). Specifically, the lumbar coronal range of motion was the most discriminative in the laboratory (p = 0.010; AUC = 0.775 [0.589–0.961]) whereas the foot-strike angle was in daily life (p = 0.001; AUC = 0.838 [0.697–0.978]). The foot-strike angle was the second best discriminative in the laboratory (p = 0.011; AUC = 0.767 [0.578–0.955]) whereas gait speed was in daily life (p = 0.003; AUC = 0.804 [0.632–0.976]). Furthermore, more daily life (n = 2) than laboratory (n = 0) measures discriminated gait in the PD group from the PD-Ctl group, after the Bonferroni’s correction.

**Table 3:** Comparison of gait measures between PD and control groups in the laboratory and during daily life

Gait measures	Laboratory					Daily Life				
	PD-Ctl (N = 15)		PD (N = 16)		Wilcox *p-*value	PD-Ctl (N = 15)		PD (N = 16)		Wilcox *p-*value
	Mean	SD	Mean	SD		Mean	SD	Mean	SD	
Cadence (steps/min)	114.49	9.65	116.94	7.67	0.446	101.37	8.07	104	11.1	0.446
Double Support (%)	21.88	2.88	20.41	3.76	0.151	27.18	5.67	27.39	5.04	0.922
Elevation at Mid Swing (cm)	0.95	0.61	1.05	0.6	0.693	3.29	0.99	3.29	0.9	0.800
Gait Speed (m/s)	1.07	0.15	1.05	0.15	0.828	*0.89*	*0.13*	*0.73*	*0.15*	*0.003*^*†*^
Foot Strike Angle (degrees)	*16.94*	*5.27*	*12.81*	*4.56*	*0.011*	*17.67*	*3.52*	*11.57*	*5.09*	*0.001*^*†*^
Toe Off Angle (degrees)	33.8	5.3	31.49	5.05	0.140	25.4	3.73	22.4	4.67	0.027
Stride Duration (s)	1.06	0.09	1.03	0.07	0.452	1.23	0.11	1.21	0.13	0.495
Stride Length (m)	1.12	0.13	1.08	0.16	0.553	*1.04*	*0.12*	*0.85*	*0.18*	*0.006*
Swing (%)	38.99	1.39	39.67	1.95	0.151	37.04	1.87	36.81	2.23	0.830
Toe Out Angle (degrees)	8.66	7.5	9.31	8.51	1.000	19.63	9.53	17.02	8.8	0.495
Lumbar—Coronal ROM (degrees)	*7.85*	*2.11*	*5.84*	*2.44*	*0.010*	6.31	1.72	5.16	1.72	0.142
Lumbar—Sagittal ROM (degrees)	5.81	2.44	5.58	2.55	0.711	6.31	1.15	6.88	1.72	0.854
Lumbar—Transverse ROM (degrees)	9.58	3.49	9.79	4.27	0.843	16.62	2.93	16.05	4.01	0.377

Italics indicates p < 0.05, and ^†^ indicates if the p-value ≤ 0.004 level of significance after Bonferroni correction (that is, 0.05/13 = 0.004)

### Laboratory versus daily life gait measures for each group

**Fig. 4:**
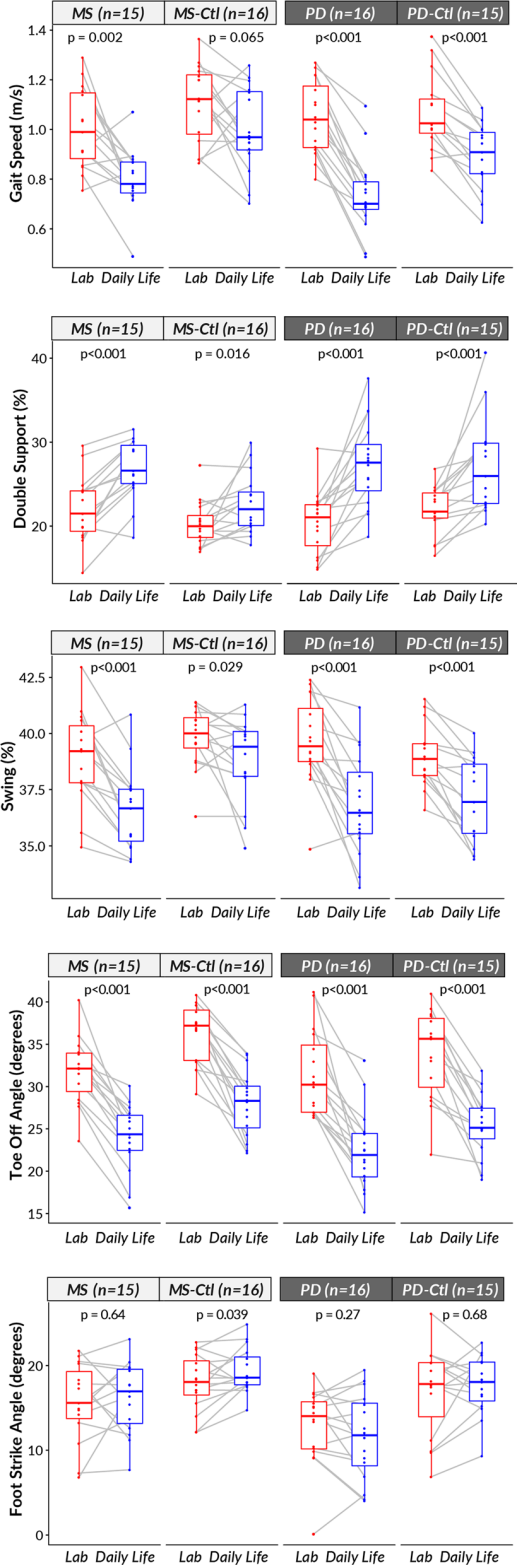
Wilcoxon paired test comparing laboratory versus daily life gait characteristics for each group (MS, MS-Ctl, PD, and PD-Ctl)

Generally, the gait characteristics in daily life compared to the laboratory reflected a slowing down behavior in all groups, and subjects performed better in the laboratory compared to daily life (see Fig. 4). Specifically, looking at the gait measures that were significant after the Bonferroni's correction in PD and MS groups, gait speed (except MS-Ctl), swing duration (% of the gait cycle), and the toe-off angle were significantly lower in daily life compared to the laboratory in all groups. Further, double support time was significantly longer in daily life compared to the laboratory in all groups. In contrast, the foot strike angle was not statistically significant between laboratory and daily life in all groups except for MS-Ctl.

## Discussion

In this study we used similar length, short walking bouts in the laboratory and daily life to investigate whether the best discriminative gait measures for PD and MS versus their respective age-matched controls remain the same in a laboratory walking test and daily life walking. Our findings demonstrated that the best measures discriminating gait characteristics in a laboratory versus daily life both in the MS and PD groups were different. Specifically, for people with MS, the toe-off angle was the most discriminative in the laboratory, whereas gait speed best discriminated in daily life. For people with PD, the lumbar coronal range of motion was the most discriminative in the laboratory (although not significant after the Bonferroni’s correction), whereas foot- strike angle best discriminated in daily life.

Although the gait measures discriminating MS and PD gait characteristics from their age-matched control groups were different, we observed an increased in the ability to discriminate neurological from control groups (i.e., AUC) for daily life gait measures compared to laboratory gait measures. All groups showed improved walking characteristics in the laboratory test compared to daily life, even though we controlled for bout length, unlike previous studies [5, 14]. For example, the gait speed was significantly higher in laboratory compared to daily life for all groups suggesting that the laboratory walking while observed may be due to the Hawthorne effect or to the lack of distractions and complexity of the environment [5]. Interestingly, the difference between the laboratory and daily life gait measures were the largest (for example, gait speed, and percentage of double support during the gait cycle) for people with PD. The large deterioration in gait characteristics during daily life suggests either that people with PD have a stronger white coat effect than the other groups, or that their gait is more impaired by challenges in daily life, such as distractions to attention, clutter, etc.

### Gait in people with MS

Long double-support time, slow gait speed, and short swing time (all affected by balance impairment and fatigue) [7, 9, 52] were significantly different daily life gait measures in MS from MS-Ctl. Indeed, gait speed double-support time and swing time as a percent of the gait cycle all discriminated gait in people with MS from gait in healthy control people over a week of daily life with a similar, excellent area under the curve [35]. In contrast, in the laboratory, the toe-off angle was the only laboratory gait measure that discriminated our mild-moderate MS from MS-Ctl group during comfortable-pace gait after Bonferroni’s correction for 13 gait characteristics. This result is consistent with our previous report of a small toe-off angle in a separate group of people with MS during a 2-min walk in the laboratory [53]. The toe-off angle is a surrogate for the push-off phase of gait produced by the power in the gastrocnemius-soleus complex, responsible for stride length and gait speed.

### Gait in people with PD

Slow gait speed (representing hypokinetic gait) and small foot strike angle (representing shuffling of gait) were significantly different daily life gait measures in the PD group compared to the PD-Ctl group. Previous studies of gait in daily life agree that foot strike angle [35], and gait speed [14] discriminated gait in PD from healthy control groups. Surprisingly, none of the laboratory gait measures discriminated gait characteristics in mild-moderate PD (ON state), from the PD-Ctl group, after Bonferroni’s correction, suggesting that monitoring gait during daily life is more sensitive to impairments from PD than gait test in the laboratory. The participants with PD showed much larger changes in their gait parameters between the laboratory and daily life than the controls or people with MS. This difference in performance in a laboratory test and daily life in people with PD may be due to their reliance on less automatic, more attention demanding gait mechanisms that would make gait in daily life more challenging [54]. The difference could also be due to people with PD being more prone to placebo effects and white coat effects than the other groups, so they perform better when their performance is observed. Alternatively, it might be that we picked up the ON and OFF fluctuations during daily life that influenced the averaged gait measures over a week. Nevertheless, assessing mobility during daily life resulted in more sensitive and specific differences in gait characteristics than laboratory gait between the PD and control groups.

### Trunk control during gait

Interestingly, the lumbar coronal range of motion was one of the top gait measures discriminating both the MS and PD groups from their age-matched controls in the laboratory, but not in daily life. The inability of lumbar motion to discriminate during daily life might be due to lumbar sensor measures being affected by the exact location of the sensor. In the laboratory, the researchers make sure the lumbar sensor location is consistent and stays securely attached throughout the testing, for all subjects, but it is hard to maintain a consistent sensor location placed by the subject in daily life conditions, and thus might not a reliable measure during daily life. Reduced lumbar range of motion while walking may reflect axial rigidity and loss of arm swing in the PD group [55], and may reflect the compensatory strategy to truncal ataxia in the MS group. In contrast to the reduced lumbar range of motion in the MS group here, our previous study found an excessive lumbar motion in people with very early MS who had normal gait speed [52].

### Bout length

Longer bout lengths, such as in 1-min laboratory tests, are known to result in faster gait speed and other accompanying measures [5, 14]. There are various ways to measure the bout length. Researchers have used bout duration [5, 14, 31], and the distance traveled during a particular walk test [32] as bout length measures. We chose to define bout length in terms of a number of strides in the bout because it helps to eliminate the effect of gait speed, per se, on the bout length. Most gait bouts during daily life have < 15 strides in all 4 groups so the 7 m × 2 in the ISAW test reflected the most common bout lengths people actually take during daily life.

### Clinical implication

Our results suggest that clinicians should consider quantitative daily life gait behavior as an integral part of a functional clinical assessment. Furthermore, this study provides encouraging results to support the use of instrumented socks for daily life gait evaluation in people with PD and MS, and also a potential to use in clinical trials, with a possibility that fewer subjects will be required for clinical trials using this quantitative measurement of mobility in daily life.

### Limitations

There are several limitations of the current study. First, we had a modest sample size of only 15–16 subjects in each group. This also resulted in a modest statistical power for detecting differences. If a larger number of subjects had been included, additional measures would have been able to discriminate between the neurological groups from their matched controls. Further analysis is needed with larger cohorts to test the generalizability of the findings. Second, we used a conservative correction for multiple hypothesis tests. Many of the tests we performed were on measures of gait that at correlated and not statistically independent. The Bonferroni correction assumes these tests are independent, so the correction may have reduced the power of the statistical tests so that additional measures are actually statistically significant. Thirdly, for daily life data, we assumed that subjects attempt to be in the ON-medication state most of the time, and hence, we compared with laboratory walking test only with subjects with their ON state. Further, future studies need to determine the test–retest reliability and sensitivity of the top mobility measures to a treatment and disease progression in daily life to be useful as digital biomarkers for clinical trials. Finally, with larger cohorts, we can investigate if the paired ROCs in a laboratory and daily life are statistically significant.

## Conclusions

Subjects, especially people with PD, generally showed better gait characteristics when observed in the laboratory compared to over a week of daily life. Different types of gait characteristics discriminated PD gait or MS gait from their age-matched controls in the laboratory versus daily life. PD and MS gait differed from each other, so clinical trials need to identify the specific gait impairments most sensitive for each neurological disease.

## Supplementary information

**Additional file 1. Table S1.** Gait measures and definitions.

## Data Availability

The data that support the findings of this study are available from the corresponding author upon reasonable request.
