# Supplementary material for: Laboratory versus daily life gait characteristics in patients with multiple sclerosis, Parkinson’s disease, and matched controls
Source: J Neuroeng Rehabil. 2020 Dec 1;17:159. doi: 10.1186/s12984-020-00781-4 (PMC7708140; doi:10.1186/s12984-020-00781-4)
Supplement: Supplementary file 1 — Additional file 1. Table S1. Gait measures and definitions. [file 12984_2020_781_MOESM1_ESM.docx]

**Laboratory versus daily life gait characteristics in patients with multiple sclerosis, Parkinson’s disease, and matched controls**

*Vrutangkumar V. Shah, PhD,^1*^ James McNames, PhD,^2,3^ Martina Mancini, PhD,^1^*

***Patricia Carlson-Kuhta, PhD,^1^*** *Rebecca I. Spain, MD, MSPH,^1,4^* *John G. Nutt, MD,^1^*

*Mahmoud El-Gohary, PhD^3^ Carolin Curtze, PhD,^5^ Fay B. Horak, PhD, PT ^1,3^*

**Table S1. Gait measures and definitions.**

| **Gait Measures** | **Definition** |
| --- | --- |
| Gait Speed (m/s) | The forward speed of the subject, measured as the forward distance traveled during the gait cycle divided by the stride duration. |
| Stride Duration (s) | The duration from the time of initial contact to the time of the next initial contact of the same foot. |
| Stride Length (m) | The forward distance traveled by the foot during a gait cycle. |
| Double Support (%) | The portion of the stride duration in which both feet are in contact with the ground, calculated as the sum of initial and terminal double support as a percentage of the stride duration. |
| Swing (%) | The portion of the stride duration in which the foot is not in contact with the ground, calculated as the period from the time of toe off until the time of initial contact as a percentage of the stride duration. |
| Cadence (steps/min) | The number of steps per minute |
| Elevation at Mid  Swing (cm) | The height of the foot sensor at the moment of maximum forward velocity of the foot, relative to the sensor height during stance. |
| Toe-Off Angle (degrees) | The angle of the foot relative to a level, horizontal surface at the time the foot leaves the ground. |
| Toe-Out Angle (degrees) | The lateral angle of the foot during the stance phase, relative to the forward motion of the gait cycle. Positive angle is outward rotation. |
| Foot Strike Angle (degrees) | The angle of the foot relative to a level, horizontal surface at the time the foot begins contact with the ground. |
| Lumbar - Transverse Range  of Motion (degrees) | The angular range torso movement at the lumbar level in the transverse plane. |
| Lumbar - Sagittal Range of  Motion (degrees) | The angular range of torso movement at the lumbar level in the sagittal plane. |
| Lumbar - Coronal Range of  Motion (degrees) | The angular range of torso movement at the lumbar level in the coronal plane. |
